# RNA-Seq Analysis Demonstrates Different Strategies Employed by Tiger Nuts (*Cyperus esculentus* L.) in Response to Drought Stress

**DOI:** 10.3390/life12071051

**Published:** 2022-07-14

**Authors:** Zhongsheng Mu, Zunmiao Wei, Jiayao Liu, Yan Cheng, Yu Song, Hongbing Yao, Xiankai Yuan, Shukun Wang, Yanhua Gu, Jingwen Zhong, Kexin Liu, Caihua Li, Jidao Du, Qi Zhang

**Affiliations:** 1 Legume Crop Laboratory, Agriculture College, Heilongjiang Bayi Agricultural University, Daqing 163319, China; muzs@163.com (Z.M.); songyu20020404@foxmail.com (Y.S.); y2260426091@163.com (H.Y.); kk13352566911@163.com (X.Y.); wskiyo@126.com (S.W.); g2324670397@163.com (Y.G.); zhongjingwen2022@163.com (J.Z.); lkx3571826659@163.com (K.L.); 2 Economic Crop Research Laboratory, Economic Crops Institute, Jilin Academy of Agricultural Sciences, Changchun 130033, China; miaomiao0825@126.com (Z.W.); jiayaoliu@yeah.net (J.L.); chengyan199910@163.com (Y.C.); licaihua_70@163.com (C.L.); 3 National Cereals Technology Engineering Research Center, Daqing 163319, China

**Keywords:** tiger nuts, drought stress, phenylpropanoid biosynthesis, starch and sucrose metabolism, ABA

## Abstract

Drought stress, an important abiotic stress, has affected global agricultural production by limiting the yield and the quality of crops. Tiger nuts (*Cyperus esculentus* L.) are C4 crops in the Cyperaceae family, which have high-quality wholesome ingredients. However, data on mechanisms underlying the response of tiger nuts to drought stress are few. Here, the variety of Jisha 1 and 15% polyethylene glycol (PEG; a drought stress simulator) were used to study the mechanisms of stress response in tiger nuts. Our evaluation of the changes in physiological indicators such as electrolyte leakage (El), malondialdehyde (MDA), hydrogen peroxide (H_2_O_2_), superoxide anion (O_2_^−^) and activities of reactive oxygen species (ROS) showed that 12 h was the most suitable time point to harvest and analyze the response to drought stress. Thereafter, we performed transcriptome (RNA-Seq) analysis in the control (CK) and stress treatment groups and showed that there was a total of 1760 differentially expressed genes (DEGs). Gene Ontology (GO) analysis showed that the DEGs were enriched in abscisic acid (ABA) terms, and pathways such as starch and sucrose metabolism (ko00500), phenylpropanoid biosynthesis (ko00940) and plant hormone signal transduction (ko04075) were significantly enriched in the Kyoto Encyclopedia of Genes and Genomes (KEGG) analysis. In addition, quantitative real-time PCR (qRT-PCR) analysis of the DEGs demonstrated an upregulation of ABA and lignin content, as well as enzyme activities in enriched pathways, which validated the RNA-Seq data. These results revealed the pathways and mechanisms adopted by the tiger nuts in response to drought stress.

## 1. Introduction

Tiger nut (*Cyperus esculentus*) is a C4 plant of the Cyperaceae family, which has a high yield and contains underground tubers with high nutrient value [1]. The tiger nut crop is widespread in tropical, temperate, and cooler zones [2] and originated from the Mediterranean area. It was domesticated and utilized as an important food source in Ancient Egypt [3,4]. The underground tubers of the tiger nuts do not only have a huge amount of nutrients, including starch, oil, sugars, protein, dietary fibers, vitamins C and E and minerals [4,5], but bioactive substances, such as phytosterols, alkaloids, saponins, tannins, flavonoids and terpenoids, as well [6,7]. These ingredients have beneficial effects in patients with diabetes, cardiovascular disease and obesity [7,8]. Since the tiger nut is an ideal foodstuff for children and seniors [9], it has become a crop of immense interest. Although tiger nuts are widely cultivated, there is limited research data on its biology [10]. Abiotic stress is one of the most important factors that may affect tiger nut yield [8].

Drought stress is an important abiotic stress and a key limiting factor which negatively affect crop productivity, owing to the global water shortage [11]. Drought stress causes significant losses in agricultural productivity, desertification, soil erosion and ecological degradation [12]. In addition, drought stress contributes to changes in the physiology and metabolism of plants, which lead to substantial reductions in the yield [13]. Drought stress can suppress the leaf area and the total chlorophyll content, produce reactive oxygen species (ROS), make leaves wilt and restrict root growth [14,15]. In addition, drought stress can also modulate activities of many oxidation-protective enzymes such as peroxidase (POD), superoxide dismutase (SOD), catalase (CAT) and ascorbate peroxidase (APX) [16], as well as the expression of some genes whose functions are associated with stress [17]. On the other hand, drought stress accumulates abscisic acid (ABA) in plants, which regulates stomatal closure and defends plants from stress [18]. ABA could induce the starch and sucrose metabolism to enhance stress tolerance [19]. Although drought stress affects all plant growth and development stages, the bud stage is the most sensitive and affects subsequent plant growth periods [20].

RNA-Seq analysis is a new analytical tool [21], which has become a method of choice because of its ability to perform more in-depth data analysis and because it can examine the whole genome [22]. Previous studies have used RNA-Seq analysis to obtain precise transcriptome information under abiotic stresses [23,24], and demonstrated some mechanisms of response to drought in species such as soybean (*Glycine max*) [25], chickpea (*Cicer arietinum*) [26], lentil (*Lens culinaris*) [27] and wheat (*Triticum aestivum*) [28]. However, data on the mechanisms of response of tiger nut (*Cyperus esculentus*) to drought stress remain scant. Here, we assessed physiological indicators such as MDA, H_2_O_2_, O_2_^−^, El and ROS activities (including SOD, POD, CAT and APX) to define the suitable time for analysis, and then employed RNA-Seq analysis to evaluate the mechanisms adopted by the tiger nuts in responding to drought.

## 2. Materials and Methods

### 2.1. Plant Materials and Treatments

The “Jisha 1” plant material was provided by the Economic Botany Institute of Jilin Academy of Agricultural Sciences. Plump seeds were sterilized with 10% NaClO for 5 min and rinsed with distilled water five times. Then, the seeds were placed in a 30 °C incubator without light for five days for cultivation. The consistent growth sprouts were selected as plant materials for further experiments. A 15% PEG concentration (the solvent was distilled water) was used to simulate drought stress (D), while distilled water treatment was set as control treatment (CK) [29]. The samples were collected after 0 h, 3 h, 6 h, 12 h, 24 h and 48 h of treatment, respectively. Ten sprouts in the same treatment were deemed as an experimental unit. The experiments were replicated five times.

### 2.2. Analysis of Physiological Indicators

Samples of 0.5 g of fresh roots were used to test the trends of the physiological indicators, which were associated with abiotic stress in plants. Electrolyte leakage (El) of samples was tested using the methods of Sutinen [30], while the malondialdehyde (MDA) of each sample was measured using the ELISA Kit (M0106, Michy, Suzhou, China) following the manufacturer’s instructions; Hydrogen peroxide (H_2_O_2_) concentration was tested using ELISA Kit (M0107, Michy, Suzhou, China) following the manufacturer’s instructions [31]; and superoxide anion (O_2_^−^) was measured using ELISA Kit (M0114, Michy, Suzhou, China) following the manufacturer’s instructions. All of these physiological indicators had five replicates.

In addition, 0.5 g fresh roots samples were used to test the trends of the activities in the ROS system. The activities of POD, SOD, CAT and APX were extracted by ELISA Kits. The activities of POD were determined using ELISA Kit (M0105, Michy, Suzhou, China) following the manufacturer’s instructions; the activities of SOD were tested using ELISA Kit (M0101, Michy, Suzhou, China) following the manufacturer’s instructions; the activities of CAT were measured using ELISA Kit (M0103, Michy, Suzhou, China) following the manufacturer’s instructions; and the activities of APX were determined using ELISA Kit (M0403, Michy, Suzhou, China) following the manufacturer’s instructions. The activities of these enzymes were analyzed in five replicates while the data of the activities were read by a microplate reader (SpectraMax^®^ 190, Molecular Devices, Los Angeles, CA, USA).

### 2.3. RNA-Seq Analysis

The tender roots at the bud stage under two treatments for 12 h were used as samples for RNA-Seq analysis, and each treatment had three biological replicates. These samples were rapidly frozen in liquid nitrogen. And then RNA was extracted by RNApure Plant Kit (CW0559, CWBIO, Beijing, China) following the manufacturer’s instructions. The RNA was measured in 1% agarose gel electrophoresis and NanoDrop instrumentation (OneC, Thermo, Waltham, MA, USA) using the methods of Zhang [20], which determined whether the quality of the RNA was qualified. The qualified RNA of these samples underwent RNA-Seq by Bio-maker (Beijing, China), while those with no reference genome were analyzed by Biocloud (https://international.biocloud.net/, accessed on 1 January 2022). UniGene was analyzed by Trinity software [32], while the differentially expressed genes (DEGs) were assessed by DESeq2 software [33]. In addition, Gene Ontology (GO) and Kyoto Encyclopedia of Genes and Genomes (KEGG) databases were used for gene and pathway enrichment analyses [34].

### 2.4. QRT-PCR Analysis

The extracted RNA in RNA-Seq was also used to synthesize single-strand cDNA using *Evo M-MLV* RT Premix for qPCR (AG11706, Accurate Biology, Hunan, China). The qRT-PCR was performed in a Light Cycler 480II system (Roche, Roche Diagnostics, Basel, Switzerland) using Hieff UNICON^®^ Universal Blue qPCR SYBR Green Master Mix (11184E, Yeasen, Shanghai, China). The reversed transcription products were diluted 10-fold and then used for qRT-PCR in a 20 µL reaction volume. While the qRT-PCR conditions were used according to the manufacturer’s instructions (Table S1). Primers for DEGs were designed by the NCBI database, and *CeUCE2* was used as reference for the qRT-PCR [34] analysis (Table S2). The relative expression levels of the selected DEGs were calculated with biological and technological replicates, using the 2^−ΔCt^ method [17].

### 2.5. Validation of the Physiological Indicators in Enriched Pathways

The ABA content was determined by HPLC-MS/MS (AB SCIEX, shimadzuLc-20AD and AB5500, Framingham, MA, USA) at the Quality Inspection Center (Dalian, China) [35]. The physiological indicators (such as starch, sucrose and lignin) [36] and the enzyme activities [37] in the enriched pathways were compared between the control and stress treatment groups and were determined by ELISA Kit (Michy, Suzhou, China) with five replicates and a microplate reader. Data were analyzed using SPSS19.0 (Armonk, NY, USA), and a *p* < 0.05 was used to demonstrate statistical significance.

## 3. Results

### 3.1. The Physiological Trends under Stress

**Figure 1:**
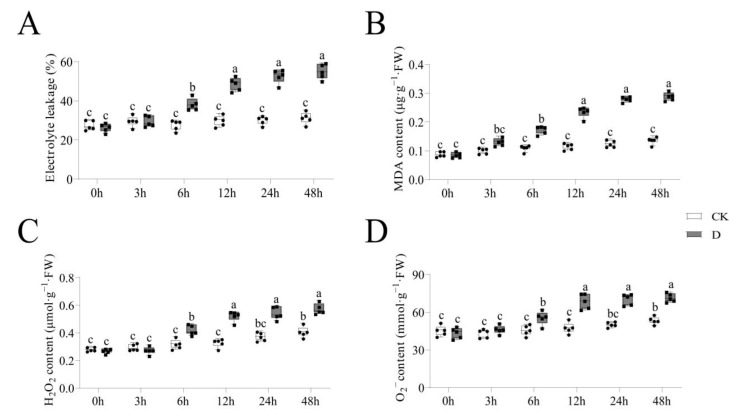
The indicators in membrane lipid peroxidation analysis at different time points under drought stress. The grey columns with black squares represent drought stress, while white columns with black circles represent CK treatment. The different lower-case letters above the columns denote significant differences (α = 0.05, LSD). (**A**) The trends of electrolyte leakage (El); (**B**) The trends of malondialdehyde (MDA); (**C**) The trends of electrolyte hydrogen peroxide (H_2_O_2_); (**D**) The trends of superoxide anion (O_2_^−^).

The roots of tiger nuts at the bud stage under control (CK) and drought stress were analyzed for the 0 h, 3 h, 6 h, 12 h, 24 h and 48 h time points. The membrane lipid peroxidation indicators such as El, MDA, H_2_O_2_ and O_2_^−^ were tested for stress trends. The results showed that the four indicators had increased significantly under drought stress compared with CK between 3 h and 12 h, but growth slowed after 12 h (Figure 1A–D). These findings illustrated that 12 h was the optimum time point to harvest and analyze the mechanisms of stress response by the plants.

**Figure 2:**
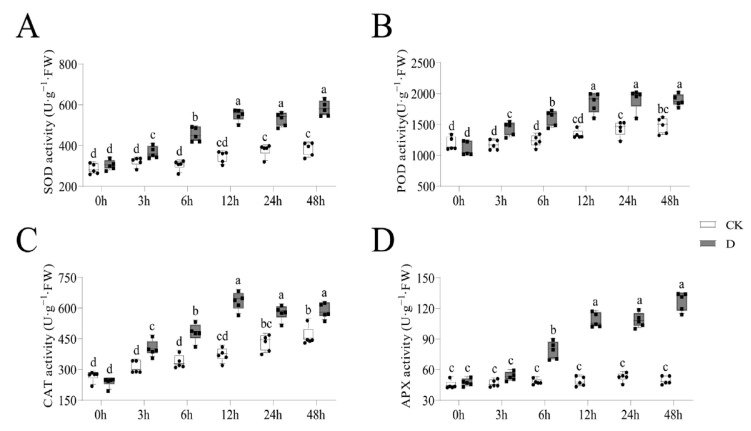
The indicators in reactive oxygen species (ROS) analysis at different time points under drought stress. The grey columns with black squares represent drought stress while white columns with black circles demonstrate CK treatment. The different lower-case letters above the columns show significant differences (α = 0.05, LSD). (**A**) The activity profile of superoxide dismutase (SOD); (**B**) The activity profile of peroxidase (POD); (**C**) The activity profile of electrolyte catalase (CAT); (**D**) The activity profile of ascorbate peroxidase (APX).

In addition, we determined the four enzymatic activities in the ROS (including SOD, POD, CAT and APX) at the defined sampling time points. The assessment of the activities of SOD, POD, CAT and APX showed that drought stress significantly increased the enzymes’ activities compared with CK, and the increment slowed after 12 h (Figure 2A–D). These findings were in sync with the indicators in membrane lipid peroxidation, thus demonstrating that 12 h is an optimum sampling time point for studying the mechanisms of response to drought stress.

### 3.2. RNA-Seq Analysis

There were 5.74 Gb of RNA-Seq data in each sample, while Q20 was more than 97.91% and Q30 was more than 94.04% (Table S3). The quality of the RNA-Seq data was confirmed and then used for further analysis. The data were uploaded in National Centre for Biotechnology Information (NCBI) database, with the accession number PRJNA821655 (Table S4).

**Figure 3:**
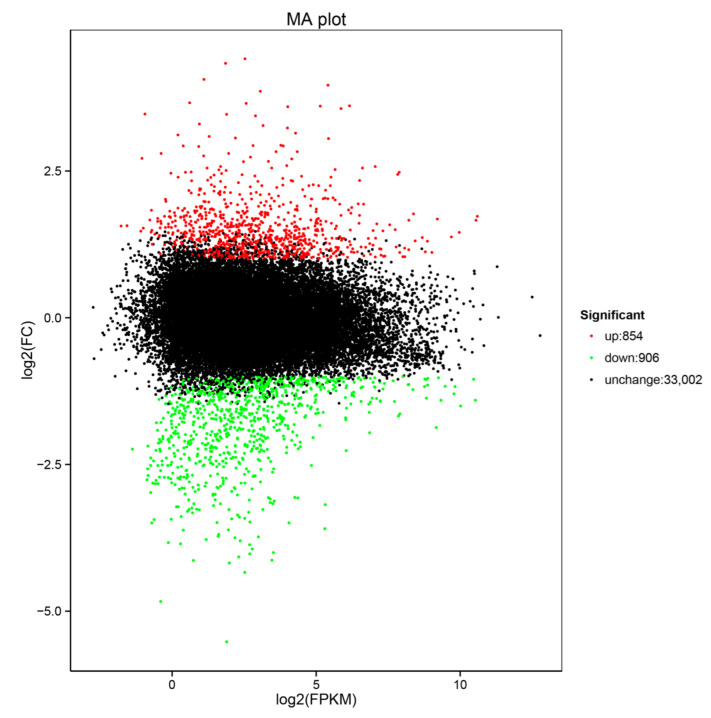
Expression plot showing RNA-Seq data. The red points show upregulated differentially expressed genes (DEGs), while the green points show downregulated DEGs.

The UniGene dataset was used as a reference for further analyses while the DEGs were assessed by DESeq2 (Table 1). Out of the total DEGs, 854 DEGs were upregulated, while 906 were downregulated (Figure 3).

### 3.3. Enrichment Analysis

**Figure 4:**
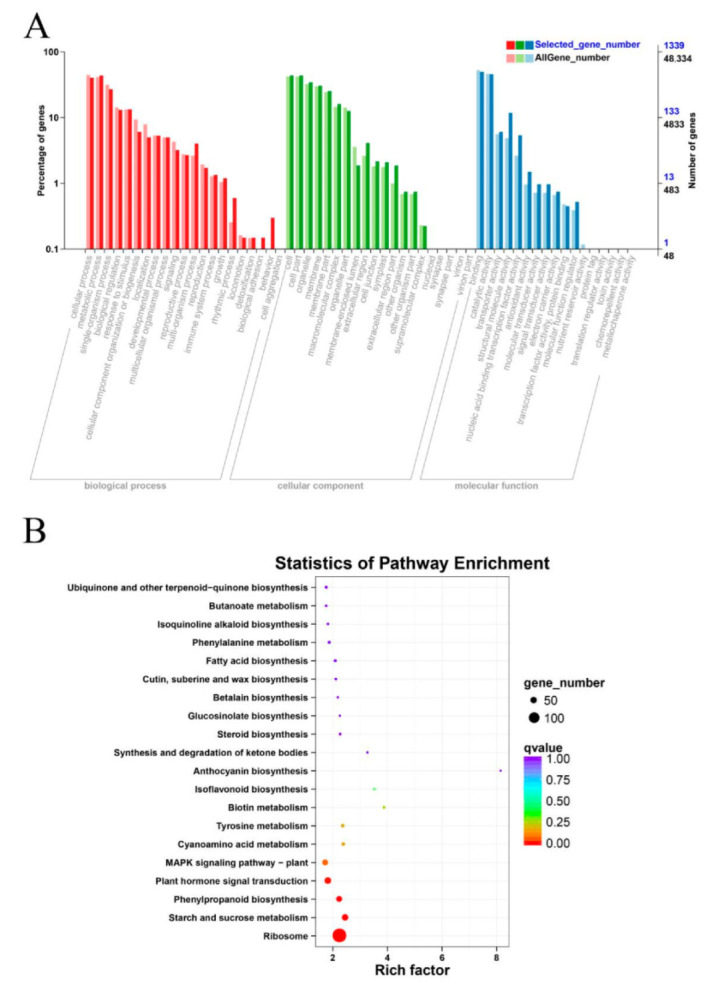
The GO and KEGG enrichment analysis of differentially expressed genes (DEGs) as shown by RNA-Seq analysis. (**A**) The GO enrichment analysis of the DEGs; the red, green and blue represent biological process, cellular component and molecular function, respectively; (**B**) The KEGG enrichment analysis of the DEGs, the circles from red to purple show the qvalues from low to high.

These DEGs underwent GO and KEGG enrichment analysis. In the GO enrichment analysis, the DEGs were enriched in three classes (Figure 4A). The *q*-value of 55 GO terms was less than 0.05, which were the significantly enriched terms, while four terms were associated with their response to ABA. These included the cellular response to abscisic acid stimulus (GO:0071215), the response to abscisic acid (GO:0009737), the cellular response to hormone stimulus (GO:0032870) and the abscisic-acid-activated signaling pathway (GO:0009738). The GO analysis demonstrated that ABA participates in responding to stress. On the other hand, the KEGG enrichment analysis showed that there was significant enrichment of DEGs in four KEGG pathways (*q*-value < 0.05), which included ribosome (ko03010), starch and sucrose metabolism (ko00500), phenylpropanoid biosynthesis (ko00940) as well as plant hormone signal transduction (ko04075) (Figure 4B). These pathways might be mediating the responses to drought stress.

### 3.4. QRT-PCR Analysis

**Figure 5:**
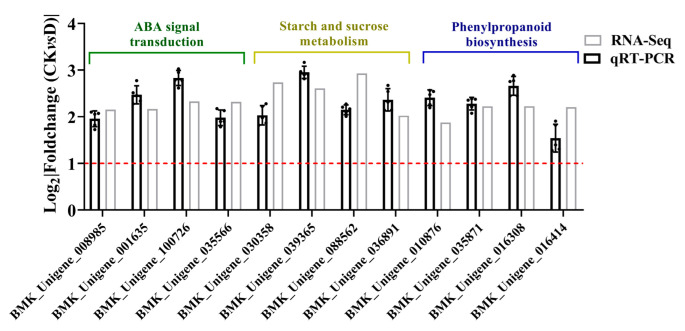
The qRT-PCR analysis of differentially expressed genes (DEGs). The gray pillars show the RNA-Seq expression analysis while the black pillars demonstrate the qRT-PCR expression profile. The green-head DEGs were enriched in abscisic acid (ABA) signal transduction pathway, the yellow-head DEGs were enriched in starch and sucrose metabolism pathway, while the blue-head DEGs were enriched in phenypropanoid biosynthesis pathway. The log_2_|Foldchange (CK vs. D)| value of more than 1 was used to denote differentially expressed genes (DEGs).

Twelve DEGs were selected for qRT-PCR analysis. The data showed that three parts of four DEGs were involved in three enriched pathways, including ABA signal transduction, starch and sucrose metabolism and phenylpropanoid biosynthesis. The qRT-PCR results showed differences in the expression compared with the RNA-Seq findings, while the trends in CK and drought stress were similar (Figure 5).

### 3.5. Enriched Pathways Analysis

**Figure 6:**
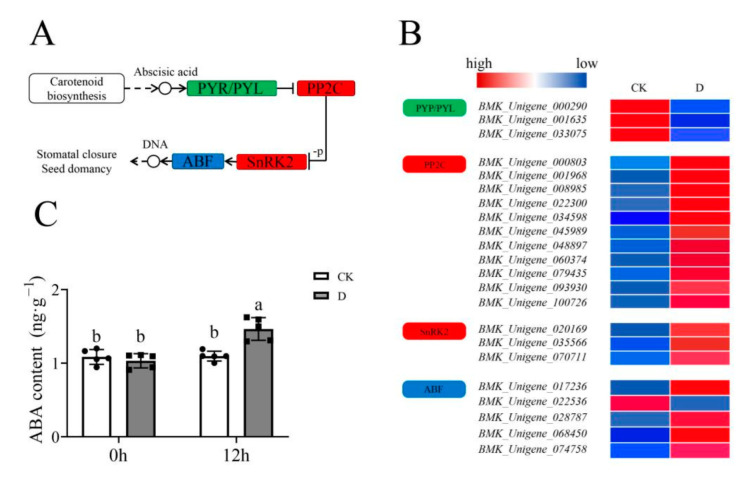
The abscisic acid (ABA) signal transduction pathway analysis in RNA-Seq. (**A**) Schematic diagram showing ABA signal transduction pathway; the red boxes represent upregulated modules, the green boxes represent downregulated modules, while the blue boxes represent upregulated and downregulated DEGs; (**B**) The expression profile of DEGs in modules enriched in pathways, the color from blue to red represent the expression from low to high; (**C**) The ABA content analysis under CK and drought treatments. The grey columns with black squares represent drought stress while white columns with black circles represent CK treatment. The different lower-case letters above the columns denote significant differences (α = 0.05, LSD).

The plant hormone signal transduction pathway (ko04075) was enriched in both the GO and KEGG analyses. Similarly, the ABA channel was also enriched (Figure 6A), together with the DEGs as shown in Figure 6B. In addition, the data showed that the ABA content had increased significantly (*p* < 0.05) under drought stress (Figure 6C), which showed that the ABA-specific pathway was enriched in response to stress.

**Figure 7:**
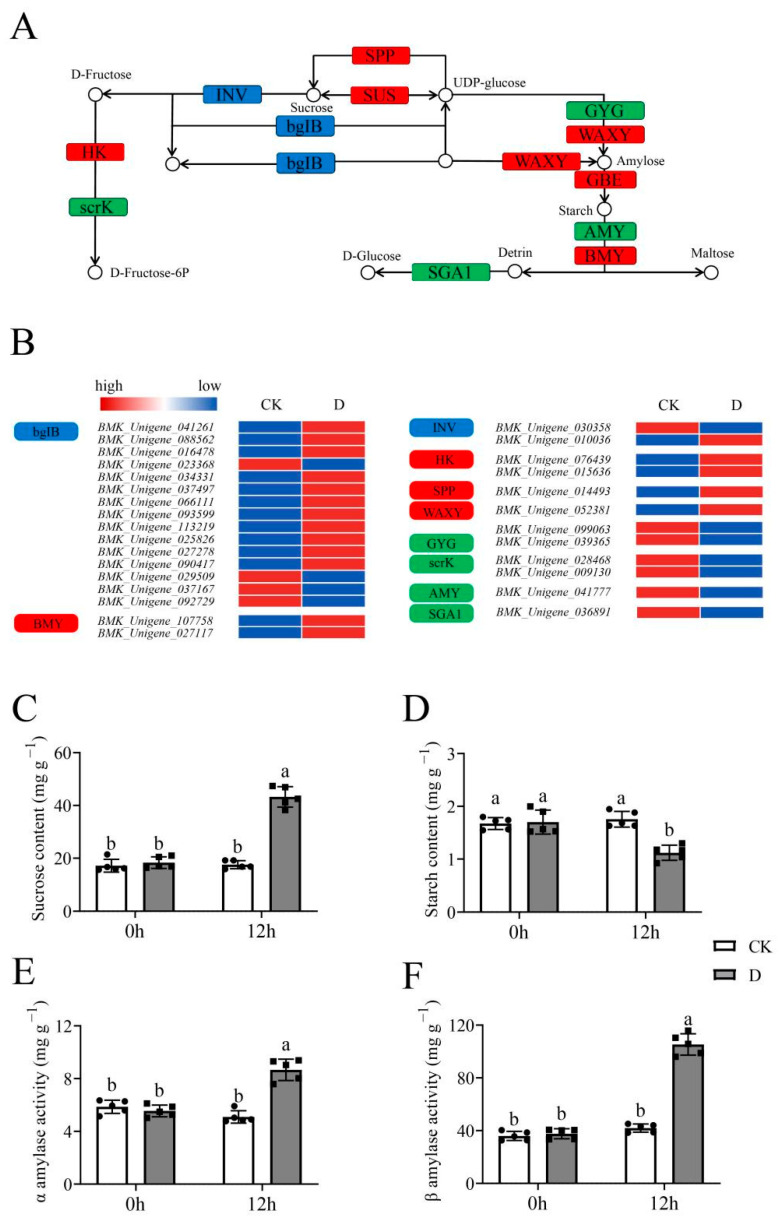
The starch and sucrose metabolism pathway analysis in RNA-Seq. The grey columns with black squares represent drought stress while white columns with black circles represent CK treatment. The different lower-case letters above the columns show significant differences (α = 0.05, LSD). (**A**) The schematic diagram of starch and sucrose metabolism pathway; the red boxes represent upregulated modules while green boxes represented downregulated modules, the blue boxes represent upregulated and downregulated DEGs contain; (**B**) The expression profile of DEGs in modules enriched in pathways, the color from blue to red represent the expression from low to high; (**C**) The sucrose content analysis under CK and drought treatments; (**D**) The starch content analysis under CK and drought treatments; (**E**) The activities of alpha-amylase analysis under CK and drought treatments; (**F**) The activities of beta-amylase analysis under CK and drought treatments.

KEGG analysis showed that the starch and sucrose metabolism pathway (ko00500) was also enriched (Figure 7A). The expression of DEGs in the ko00500 pathway was significantly changed (Figure 7B). In addition, there was an increase in the sucrose content while the starch content was suppressed with the increasing activities significantly (*p* < 0.05) of alpha-amylase and beta-amylase (Figure 7C–F). Therefore, these results revealed that the starch and sucrose metabolism pathway was involved in responses to drought stress.

**Figure 8:**
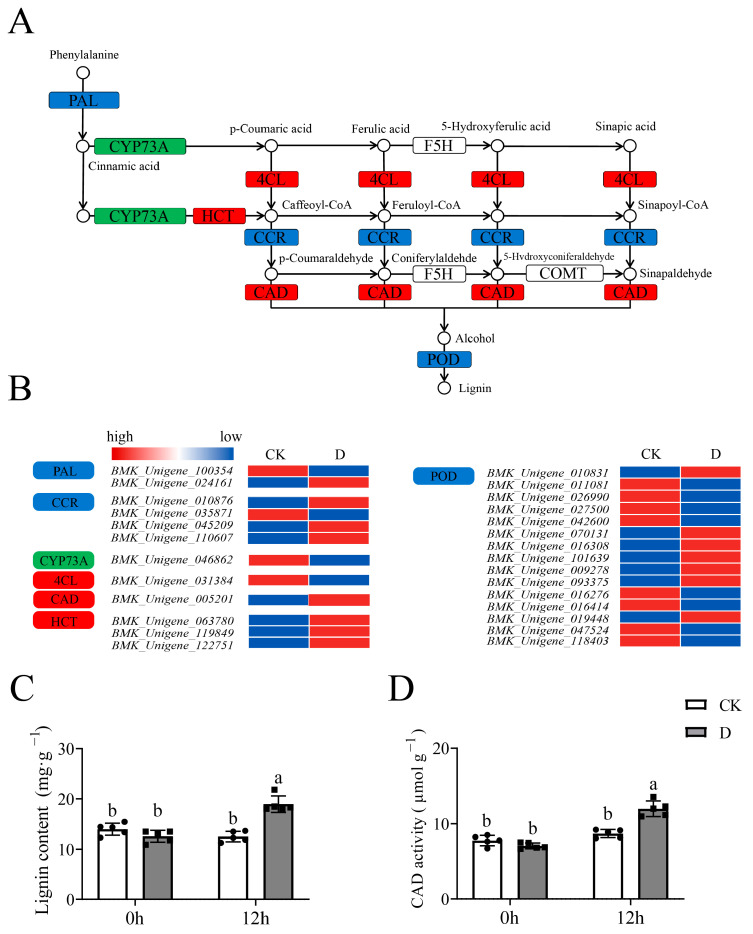
The phenypropanoid biosynthesis pathway analysis in RNA-Seq. The grey columns with black squares represent drought stress, while white columns with black circles represent CK treatment. The different lowercase letters above the columns show significant differences (α = 0.05, LSD). (**A**) Schematic diagram of phenypropanoid biosynthesis pathway, the red boxes represent upregulated modules while green boxes represented downregulated modules, the blue boxes represent upregulated and downregulated DEGs; (**B**) The expression profile of DEGs in modules enriched in pathways, the color from blue to red represent the expression from low to high; (**C**) The lignin content analysis under CK and drought treatments; (**D**) The activities of cinnamyl-alcohol dehydrogenase (CAD) analysis under CK and drought treatments.

In addition, there was enrichment in phenylpropanoid biosynthesis pathway (ko00940) (Figure 8A), and the expression of DEGs in the ko00500 pathway had significant changes (Figure 8B). Similarly, lignin content had increased significantly (*p* < 0.05) and there were significant shifts (*p* < 0.05) in the activities of cinnamyl-alcohol dehydrogenase (CAD) (Figure 8C,D). These results revealed that phenylpropanoid biosynthesis was one of the pathways that mediates responses to stress.

## 4. Discussion

The bud stage is the most sensitive stage in plants under abiotic stresses, which could affect growth in later stages [38]. Drought stress limits the growth, development and survival rate of plants, leading to tremendous losses in yield, thus threatening global agricultural production [39]. In our study, 15% PEG was used to simulate drought stress [29,40,41,42]. Therefore, the analysis of the response of sprouts to drought stress at the bud stage is important for actual agricultural production [43]. Previous studies have shown that roots can be used to study responses to drought stress in common bean (*Phaseolus vulgaris*) [44], rice (*Oryza sativa*) [45], soybean (*Glycine max*) [46] and peanut (*Arachis hypogaea*) [47]. In this study, 15% PEG was used to simulate the drought stress and roots of the tiger nuts were used as sample tissues, as previously described [48,49].

Drought-exposed plants exert different mechanisms to regulate the responses to stress [39]. A 12 h duration under drought stress has been shown to be an optimal sampling time to study stress responses [50]. Exposing plants to stress for a short time does not cause changes in the phenotype, but physiological indicators might change significantly [51]. El is an indicator which reflects the state of a plant’s membrane system [52]. El increases when plants are subjected to stress or other damaging stimuli [53]. The El was shown to increase under drought stress, but the rate of increase after 12 h was not significant. MDA content is a parameter which reflects antioxidant potential, and indirectly reflects the degree of tissue peroxidative damage [54]. There was significant increase in the MDA content under stress before 12 h, which was not observed after the 12 h time point. In addition, H_2_O_2_ and O_2_^−^ reflect the plant’s resilience under stress [55]. Our study showed that 12 h of exposure to stress increased the levels of H_2_O_2_ and O_2_^−^. On the other hand, the ROS system plays a dual role in the growth and development of plants [56], and enzyme activities directly reflect ROS in plants. In this study, the POD, SOD, CAT and APX activities increased more within 12 h of exposure. In line with previous data, these data revealed that 12 h was a suitable sampling time to study the mechanisms involved in responding to drought stress in tiger nuts [50,57,58].

**Figure 9:**
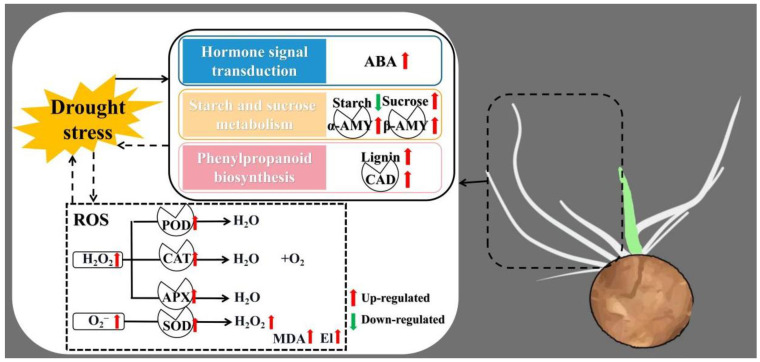
Schematic representation of the mechanisms employed by tiger nuts in response to drought stress at bud stage, where red up-arrows indicate upregulation while green arrows show downregulation.

There are many mechanisms involved in responding to abiotic stress, especially drought stress, in plants [59]. Drought stress induces ABA accumulation and triggers rapid biochemical and physiological responses [60]. The ABA pathway is a well-established pathway which has been shown to respond to drought stress [61]. In this study, DEGs were enriched in ABA-associated pathways in the GO and KEGG analyses, while the ABA content changed under drought stress, which demonstrated that ABA-associated pathways participate in responding to drought stress in tiger nuts. The starch and sucrose metabolism pathway is closely associated with ABA accumulation [62,63,64], which was also associated with responses to drought stress [36]. Starch is made in chloroplasts in leaves which the content decreased under drought stress [65], while sucrose is produced in leaves following photosynthesis, which the content increased to balance osmotic pressure within plant cells under drought stress [64]. Also in this study, the starch and sucrose metabolism pathway was an enriched pathway in the KEGG analysis, which showed an increase in the sucrose content with changes in amylase activities (α and β amylase), while the content of starch was reduced. This could be because the starch was converted to sugar, which increased osmotic pressure in responding to stress. In addition, lignin was recognized as having an important role in drought resistance [66] while the CAD enzyme was used to characterize drought tolerance [67]. Here, the phenylpropanoid biosynthesis (ko00940), as a stress-resistant pathway [68], was enriched in the KEGG analysis, while the contents of lignin and CAD activities were significantly changed (Figure 9). In addition, the three pathways have been reported to participate in responding to abiotic stress in *Hibiscus cannabinus*, *Miscanthus* and tomato (*Solanum lycopersicum*) [69,70,71]. Our study demonstrated the mechanisms underlying the drought stress responses by tiger nuts, which enhances drought tolerance.

## 5. Conclusions

Our analyses showed that 12 h was a suitable sampling time for tiger nuts when studying stress responses. A comparative analysis of the RNA-Seq data under drought stress showed a total of 1760 DEGs, where 854 genes were upregulated while 906 genes were suppressed. These DEGs were enriched in ABA-associated terms in the GO analysis, while the starch and sucrose metabolism (ko00500), phenylpropanoid biosynthesis (ko00940) and plant hormone signal transduction (ko04075) pathways were significantly enriched in the KEGG analysis. Taken together, our data revealed the mechanisms that underly the responses exerted by tiger nuts to drought stress.

## Figures and Tables

**Table 1:** Analysis of differentially expressed genes (DEGs).

Groups	Total DEGs	Up DEGs	Down DEGs
CK vs. D	1760	854	906

## Data Availability

All data are contained within the article.

## Supplementary Materials

The following supporting information can be downloaded at: https://www.mdpi.com/article/10.3390/life12071051/s1, Table S1: The qRT-PCR program used in this study; Table S2: QRT-PCR primers for differentially expressed genes (DEGs); Table S3: The quality analysis of the RNA-Seq data; Table S4: The RNA-Seq data in National Centre for Biotechnology Information (NCBI) database.
