# Supplementary material for: RNA-Seq Analysis Demonstrates Different Strategies Employed by Tiger Nuts (Cyperus esculentus L.) in Response to Drought Stress
Source: Life (Basel). 2022 Jul 14;12(7):1051. doi: 10.3390/life12071051 (PMC9322875; doi:10.3390/life12071051)
Supplement: Supplementary file 1 [file life-12-01051-s001.zip › Table S1.pdf]

Table S1. The qRT-PCR program used in this study.

Cycle steps	Temperature	Time	Number of cycles
Pre denaturation	95℃	2 min	1
Denaturation	95℃	10 sec	40
Annealing / extension	60℃	30 sec	
Melting curve stage	Instrument default settings		1
