# Supplementary material for: RNA-Seq Analysis Demonstrates Different Strategies Employed by Tiger Nuts (Cyperus esculentus L.) in Response to Drought Stress
Source: Life (Basel). 2022 Jul 14;12(7):1051. doi: 10.3390/life12071051 (PMC9322875; doi:10.3390/life12071051)
Supplement: Supplementary file 1 [file life-12-01051-s001.zip › Table S2.pdf]

Table S2. QRT-PCR primers for differentially expressed genes (DEGs).

Unigene ID	Forward primer	Reverse primer
CeUCE2	ATCATCAAGGAGACCCAGCG	CTTAGGGGCAGCCATAGGA
Unigene035871	CTGGGTGCCAAGGAGTGTT	CCTACATCAGCCGCCACAT
Unigene016414	CGGTAAAGGTTGTGGTATC	TCAGGCATTGAGTAAGGAG
Unigene010876	TGCTCAAATGGGGTGAAGTC	TCAGAGGCTGTGGAGATAAT
Unigene016308	CCAAAATCAGGACACACAT	ACTACCTCCGCTTCACCAA
Unigene030358	TTTTCGGAGCCACGAGTTTC	AGCAACACAACCCTGCGAGT
Unigene039365	TGGTATCGTCATCGGCTCT	CTGCTCGTCCAAATGTGCT
Unigene088562	TAAAAGAAGAAAAACAGGTA	TCGTCTCGGAGCAATC
Unigene036891	GTCGGCATCACACAAAGTG	TTCGGAGCTGGAGAAAGGT
Unigene001635	CAGAGGAGGATACCAAGAT	CAAAAACAAAACAAAAAAG
Unigene008985	TCCTTCCATCCTTCCTTCC	CAATCAACCAAACCCCCAA
Unigene035566	AACAATCTCTATCGGGGGA	ACGGACGAATTAAAAACCA
Unigene100726	CGCAGTTGTGGCTGTTGTC	TACCTTGTGGTCCGATGAA
