# Supplementary material for: RNA-Seq Analysis Demonstrates Different Strategies Employed by Tiger Nuts (Cyperus esculentus L.) in Response to Drought Stress
Source: Life (Basel). 2022 Jul 14;12(7):1051. doi: 10.3390/life12071051 (PMC9322875; doi:10.3390/life12071051)
Supplement: Supplementary file 1 [file life-12-01051-s001.zip › Table S3.pdf]

Table S3. The quality analysis of the RNA-Seq data.

SampleID	CK			D		
	CK1	CK2	CK3	D1	D2	D3
Total Reads	22749315 (100%)	20020682 (100%)	19237420 (100%)	20431363 (100%)	22181832 (100%)	19869883 (100%)
Mapped Reads	19201875 (84.41%)	16790187 (83.86%)	16127525 (83.83%)	17152105 (83.95%)	18663491 (84.14%)	16771789 (84.41%)
Uniq mapped Reads	9049575 (47.13%)	8003812 (47.67%)	7559717 (46.87%)	7703515 (44.91%)	8534202 (45.73%)	7749486 (46.21%)
Multi mapped Reads	10152300 (52.87%)	8786375 (52.33%)	8567808 (53.13%)	9448590 (55.09%)	10129289 (54.27%)	9022303 (53.79%)
GC(%)	48.68	48.51	48.72	47.97	48.51	48.75
Q20(%)	97.91	98.05	98.09	98.04	98.09	98.02
Q30(%)	94.04	94.52	94.62	94.44	94.64	94.46
N(%)	0	0	0	0	0	0
GC_separate(%)	1.77	1.73	1.7	1.52	1.7	1.66
GC_fluctuate(%)	2.21	2.13	2.11	2.07	2.13	2.07
GC_diff(%)	0.78	0.78	0.78	0.78	0.78	0.78
