# Supplementary material for: RNA-Seq Analysis Demonstrates Different Strategies Employed by Tiger Nuts (Cyperus esculentus L.) in Response to Drought Stress
Source: Life (Basel). 2022 Jul 14;12(7):1051. doi: 10.3390/life12071051 (PMC9322875; doi:10.3390/life12071051)
Supplement: Supplementary file 1 [file life-12-01051-s001.zip › Table S4.pdf]

Table S4. The RNA-Seq data in National Centre for Biotechnology Information (NCBI) database.

Bioproject_accession	Accession	Study	Bioproject_accession	Biosample_accession	Title
PRJNA821655	SRR18568016	SRP366968	PRJNA821655	SAMN27118664	CK1
	SRR18568015	SRP366968	PRJNA821655	SAMN27118665	CK2
	SRR18568014	SRP366968	PRJNA821655	SAMN27118666	CK3
	SRR18568013	SRP366968	PRJNA821655	SAMN27118667	D1
	SRR18568012	SRP366968	PRJNA821655	SAMN27118668	D2
	SRR18568011	SRP366968	PRJNA821655	SAMN27118669	D3
